# A case of situs inversus totalis with sigmoid volvulus: a case report of a rare coexistence

**DOI:** 10.1097/MS9.0000000000003651

**Published:** 2025-07-25

**Authors:** Shrestha Anmol Singh, Puspharaj Shrestha, Pahari Nabin, Singh Chandresh Kumar, Chhetri Sahas, Pahari Mukesh

**Affiliations:** a Department of Intensive Care, Devdaha Medical College, Rupandehi, Nepal; b Department of Emergency Medicine, Devdaha Medical College, Rupandehi, Nepal; c Department of Intensive Care, Lumbini Provincial Hospital, Rupandehi, Nepal; d Department of Surgery, Devdaha Medical College, Rupandehi, Nepal; e Devdaha Medical College, Rupandehi, Nepal

**Keywords:** case report, dextrocardia, Nepal, sigmoid volvulus, situs inversus totalis

## Abstract

**Introduction:**

Situs inversus totalis is an uncommon congenital disease characterized by the full transposition of thoracic and abdominal organs. Sigmoid volvulus, an unusual but potentially catastrophic cause of major bowel obstruction, is rarely associated with Situs inversus.

**Case presentation:**

A 76-year-old male presented with abdominal pain and obstipation for 5 days. Clinical examination and imaging revealed signs of bowel obstruction and dextrocardia. Computed tomography (CT) scan abdomen confirmed sigmoid volvulus and situs inversus totalis. Emergency laparotomy revealed a 360-degree twisted, dilated sigmoid colon. Manual detorsion and excision of fibrous bands were performed. The patient recovered uneventfully.

**Clinical discussion:**

While sigmoid volvulus is a known surgical emergency, its diagnosis in situs inversus patients can be challenging due to reversed anatomical landmarks. Imaging plays a critical role in both diagnosis and surgical planning.

**Conclusion:**

Awareness of reversed anatomy in situs inversus is crucial for prompt diagnosis and successful surgical management acute abdominal conditions like volvulus

## Introduction

Situs inversus totalis is a rare congenital disorder in which thoracic and abdominal organs are mirrored from their normal positions, with an incidence between 1 in 10 000 to 1 in 50 000 live births^[1]^. Although often asymptomatic and incidentally discovered, this anatomical variation can complicate clinical evaluation, delay diagnosis, and present significant challenges during surgical procedures^[2]^. Sigmoid volvulus refers to the torsion of the sigmoid colon around its mesentery, resulting in obstruction and potential vascular compromise. It is more commonly observed in elderly individuals, those with chronic constipation, or in populations with high-fiber diets^[3]^. The diagnosis relies on a combination of clinical suspicion and radiographic findings, with prompt intervention necessary to prevent ischemia or perforation. The coexistence of sigmoid volvulus in a patient with situs inversus totalis is exceedingly rare and presents unique diagnostic and surgical challenges due to the reversed anatomical orientation. The simultaneous occurrence of these two conditions is extremely rare and complicates diagnosis due to altered anatomical landmarks. Awareness and recognition of this condition are crucial, particularly in emergency settings, to facilitate accurate diagnosis and appropriate clinical decision-making. Early identification can help avoid diagnostic confusion, enable proper surgical planning, and reduce the risk of intra-operative and post-operative complications. Our case report has been written in line with SCARE guidelines^[4]^.

**HIGHLIGHTS**

- Situs inversus totalis is a rare congenital anomaly with complete mirror-image positioning of internal organs.
- Diagnostic imaging modalities, including abdominal and chest radiographs, computed tomography (CT), and barium studies, play a crucial role in confirming the presence of situs inversus totalis.
- Sigmoid volvulus, though uncommon, may be life-threatening and requires prompt diagnosis and intervention.
- The coexistence of situs inversus with sigmoid volvulus poses diagnostic and surgical challenges.
- CT imaging is essential for identifying both conditions and guiding surgical management.
- Early recognition and tailored surgical approaches ensure favorable outcomes in such rare presentations.

## Case presentation

A 76-year-old male presented to the emergency department with abdominal pain and inability to pass stool for 5 days. The pain was localized to the hypogastric region, insidious in onset, dull, non-radiating, and associated with nausea, vomiting, and decreased appetite. He had no history of fever, urinary symptoms, or prior abdominal surgeries and he denies any similar symptoms in the past. He had been using a salbutamol rotahaler as needed for asthma over the past 12 years.

On examination, he was moderately dehydrated with stable vitals: pulse 110 bpm, blood pressure 100/80 mmHg, respiratory rate 18 breaths/min, and SpO_2_ 96% on room air. A focused history and complete clinical examination were conducted, which took approximately 12 minutes. Abdominal examination revealed a distended, tympanic abdomen with diffuse tenderness. Liver dullness was appreciated in the left upper quadrant, and heart sounds were auscultated in the right 5th intercostal space, suggestive of dextrocardia. Bowel sounds were sluggish, and a digital rectal examination showed an empty rectum.

A provisional diagnosis of acute intestinal obstruction with possible situs inversus was made. The patient was kept nil per oral and started on intravenous fluids, pantoprazole, ceftriaxone, ornidazole, hyoscine butyl bromide, and tramadol.

**Figure 1.**
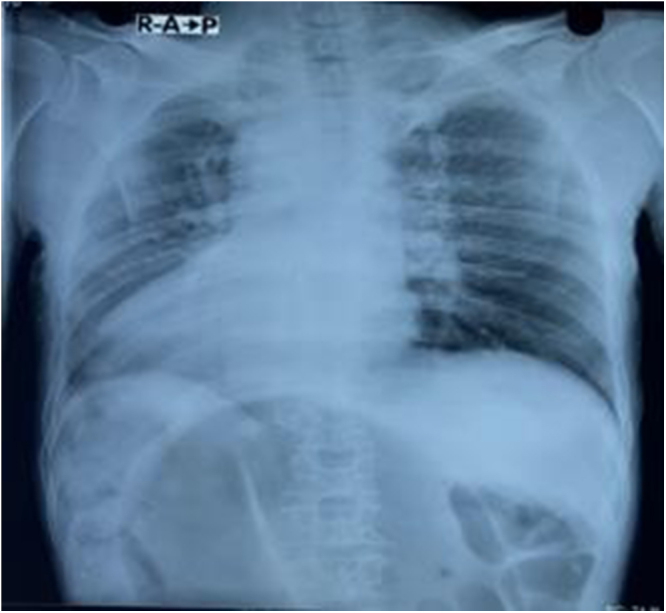
Antero-posterior view of chest showing right sided heart with hilar lymphadenopathy.

**Figure 2.**
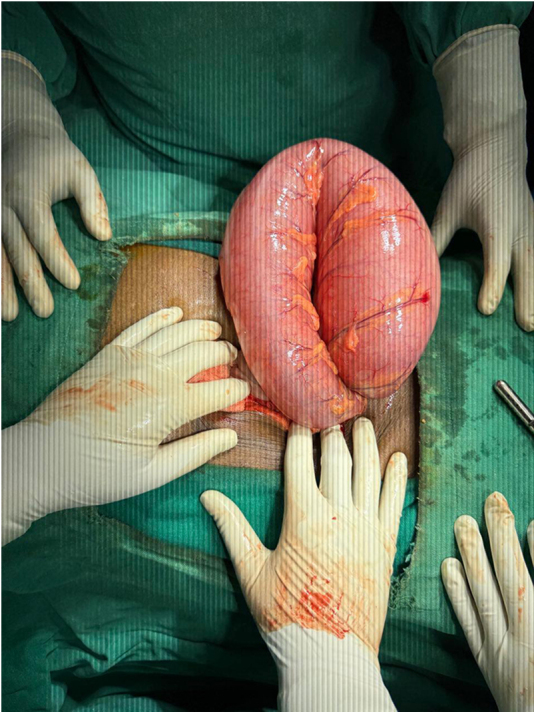
Intraoperative finding of sigmoid volvulus.

**Figure 3.**
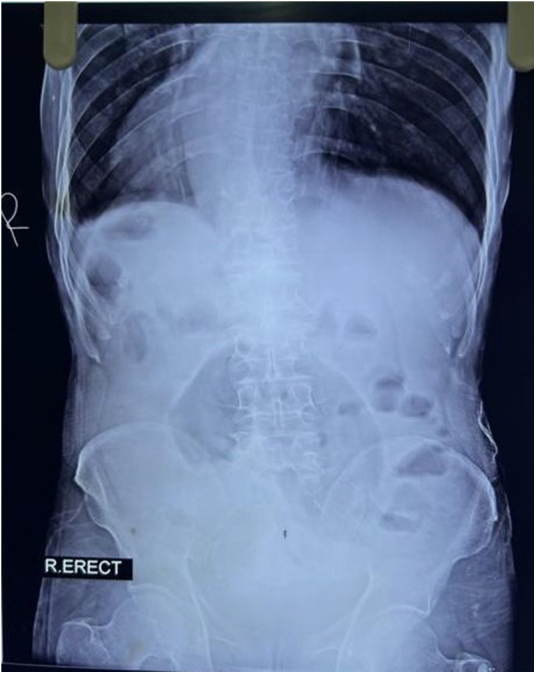
X ray abdomen (erect view) showing post-operative status of patient.

All routine laboratory investigations were completed, and reports were available within 40 minutes of patient presentation to Emergency Department. Laboratory investigations were unremarkable. A plain abdominal X-ray was order within 30 minutes which demonstrated a characteristic “coffee bean” sign. Chest X-ray showed dextrocardia (Fig. 1). Ultrasonography was done immediately after X ray which revealed a left-sided liver, right-sided spleen, cholelithiasis, a right renal cortical cyst, and left-sided mild hydronephrosis. Contrast-enhanced computed tomography (CECT) scan of the abdomen was done approximately 10 minutes after the ultrasonography which confirmed situs inversus totalis and sigmoid volvulus with a closed loop, twisted mesentery and the classic “Whirl sign.” The patient and accompanying family members were unaware about the condition and were accordingly provided detailed counseling regarding the diagnosis of Situs Inversus, including its anatomical implications and the potential challenges it may pose during surgical procedures and clinical management. The counseling process took approximately twenty minutes, as the patient and patient’s family members were unfamiliar with both of the condition, and required time to understand the nature, urgency and complications of the condition. The patient was transferred to the operation theatre approximately 90 minutes after arriving at the Emergency department. An emergency laparotomy was performed via a midline incision, which revealed a 360-degree clockwise-twisted, dilated, and edematous sigmoid colon (Fig. 2). It was found to the right of midline incision, extending toward the right iliac fossa—an anatomical variation from the typical counterclockwise rotation and usual position in left iliac fossa. Manual detorsion was performed, and fibrous bands at the twisted site were excised. No ischemia or perforation was noted. Peritoneal fluid was evacuated, and the abdomen was closed. The postoperative period was uneventful, and the patient was discharged on the 7th postoperative day with X-ray of an abdomen which is shown in Fig. 3.

The patient was scheduled for follow-up visit 7 days later. The patient underwent a thorough clinical examination to assess for any post-operative complications. The surgical site was evaluated, and the suture was removed without difficulty. The patient reported a normal appetite and satisfactory bowel function, including regular passage of stool and flatus. No new complaints were noted at the time of follow-up. Based on the clinical finding and recovery progress, the post-operative prognosis was assessed to be good.

## Clinical discussion

### Situs inversus

Situs inversus is a rare congenital anomaly with a genetic basis, which can manifest either as a partial anomaly involving the thoracic or abdominal organs (situs inversus partialis) or as a complete reversal of both thoracic and abdominal viscera (situs inversus totalis). Normally, embryonic mid-gut rotation occurs in a counterclockwise direction; however, in situs inversus totalis, this rotation is reversed, resulting in a mirror-image arrangement of the visceral organs.

While it is sometimes associated with primary ciliary dyskinesia (PCD), a genetic disorder affecting ciliary function, most SI cases occur without PCD, and their cause remains unclear. In a study of 15 individuals with SI (six with PCD and nine without), whole-genome sequencing was performed alongside 15 healthy controls. Among non-PCD SI cases, left-handedness was observed in 5 of 9, suggesting a possible developmental link between brain and organ asymmetry. All six PCD-related SI cases had mutations in known PCD genes. Interestingly, two non-PCD SI individuals also carried these mutations, indicating incomplete penetrance. Other mutations were identified in PKD1L1 and CFAP52, both associated with SI without PCD. However, five non-PCD SI cases, including three left-handers, showed no clear genetic cause, pointing to possible environmental or random developmental factors^[5].^ Patients with situs inversus totalis are often asymptomatic, with the condition typically being identified incidentally during routine health examinations or investigations for unrelated medical concerns.

### Sigmoid volvulus

Volvulus is defined as the torsion of a segment of the alimentary tract which often leads to bowel obstruction. The most common site of volvulus is the sigmoid colon and caecum**^[6-9].^** The other sites for volvulus are the gallbladder, stomach, small bowel, splenic flexure, and transverse colon which are rare in incidence. The pathophysiology behind the clinical condition is described as the twisting of an air-filled loop of the alimentary tract leading to obstruction and impairment of vascular perfusion of the affected segment.

The clinical manifestation of volvulus consists of progressive abdominal pain that is insidious in onset and associated with nausea, abdominal distention, and constipation. Due to this insidious and progressive nature of abdominal pain, the majority of patients usually present in the Emergency/Outpatient department with 4 to 5 days of history of colicky abdominal pain**^[10].^** Diagnosis of a volvulus is established by imaging. Laboratory testing is done to rule out other causes of acute abdominal pain. Abdominal X-rays are done initially to visualize any signs of bowel obstruction. Signs such as “apex of the loop under the left heme-diaphragm, inferior convergence on the left, and the left flank overlap sign is a diagnostic finding on abdominal radiograph for sigmoid volvulus**^[11].^** Contrast enema showing twisted or bird’s beak configuration is characteristic appearance of a sigmoid volvulus. Abdominal CT scan usually establishes the diagnosis of volvulus and can rule out other causes of abdominal pain. A whirl pattern, caused by the dilated sigmoid colon around its mesocolon and vessels, and a bird-beak appearance of the afferent and efferent colonic segment is diagnostic finding of sigmoid volvulus^[12].^ However, typical features may be absent in one-fourth of the CT scans**^[13].^** Other supportive features for diagnosis includes the absence of rectal gas, apparent separation of sigmoid walls by adjacent mesenteric fat due to incomplete twisting or folding. The presence of pneumatosis intestinalis, portal venous gas, of loss of bowel wall enhancement on CT scan is suggestive of bowel necrosis**^[13].^** Management depends on the presence of alarming signs of perforation and peritonitis. Patients with alarming signs should undergo immediate surgery and resection of compromised bowel in its volvulized position. Resection followed by reconstruction showed be done depending upon the extents of vascular compromise and bowel injury. Patients without alarming signs should undergo flexible sigmoidoscopy to detorse the twisted segment, and if successful, surgical resection can be done thereafter. Patients with failed endoscopic detorsion should undergo immediate surgery.

## Conclusion

This case shows the rare occurrence of sigmoid volvulus in a patient with situs inversus totalis. Although case of sigmoid volvulus in a patient with situs inversus has been recorded, a definitive association has not been established yet. This report emphasizes the importance of maintaining a high index of clinical suspicion, conducting thorough radiological evaluations, and implementing precise surgical management in anatomically complex cases. It also outlines the diagnostic challenges posed by atypical symptom presentation, which can lead to delayed diagnosis, as well as the technical difficulties encountered during surgery due to altered anatomical landmarks. Awareness of reversed anatomy is essential to ensure accurate diagnosis and improve surgical outcomes.

## Data Availability

The dissemination of the article data is freely accessed.
